# Simultaneous Administration of Hyperbaric Oxygen Therapy and Antioxidant Supplementation with *Filipendula ulmaria* Extract in the Treatment of Thermal Skin Injuries Alters Nociceptive Signalling and Wound Healing

**DOI:** 10.3390/medicina59091676

**Published:** 2023-09-17

**Authors:** Milos Krstic, Nemanja Jovicic, Dragica Selakovic, Bojana Krstic, Natalija Arsenijevic, Milica Vasiljevic, Pavle Milanovic, Jovana Milanovic, Dragan Milovanovic, Marko Simic, Jelena S. Katanic Stankovic, Gvozden Rosic

**Affiliations:** 1 Department of Physiology, Faculty of Medical Sciences, University of Kragujevac, 34000 Kragujevac, Serbia; krsticmilos@hotmail.rs (M.K.); dragica984@gmail.com (D.S.); b.barlov_92@hotmail.com (B.K.); markosimicderm@gmail.com (M.S.); grosic@medf.kg.ac.rs (G.R.); 2 Department of Histology and Embryology, Faculty of Medical Sciences, University of Kragujevac, 34000 Kragujevac, Serbia; 3 Department of Dentistry, Faculty of Medical Sciences, University of Kragujevac, 34000 Kragujevac, Serbia; arsenijevicnatalija@gmail.com (N.A.); milicavaska13@gmail.com (M.V.); pavle11@yahoo.com (P.M.); jovannakg94@gmail.com (J.M.); 4 Clinical Pharmacology Department, Clinical Centre Kragujevac, 34000 Kragujevac, Serbia; piki@medf.kg.ac.rs; 5 Department of Pharmacology and Toxicology, Faculty of Medical Sciences, University of Kragujevac, 34000 Kragujevac, Serbia; 6 Department of Science, Institute for Information Technologies Kragujevac, University of Kragujevac, 34000 Kragujevac, Serbia; jkatanic@kg.ac.rs

**Keywords:** thermal skin injury, nociception, hyperbaric oxygen therapy, antioxidant supplementation, rats

## Abstract

*Background and Objectives*: Thermal skin injuries are a prevalent cause of skin damage, potentially leading to severe morbidity and significant mortality. In this study, we intended to estimate the effects of HBO (hyperbaric oxygen treatment) and antioxidant supplementation with *Filipendula ulmaria* extract, individually and simultaneously, in the treatment of thermal skin injuries. *Materials and Methods*: As a thermal skin injury experimental model, we used two-month-old male Wistar albino rats. Thermal injuries were made with a solid aluminium bar at a constant temperature of 75 °C for 15 s. Hyperbaric oxygen treatment was performed in a specially constructed hyperbaric chamber for rats (HYB-C 300) for seven consecutive days (100% O_2_ at 2.5 ATA for 60 min). Antioxidant supplementation was performed with oral administration of *Filipendula ulmaria* extract dissolved in tap water to reach a final concentration of 100 mg/kg b.w. for seven consecutive days. *Results:* Simultaneous administration of hyperbaric oxygen therapy and antioxidant supplementation with *Filipendula ulmaria* extract significantly ameliorated the macroscopic and histopathological characteristics of the wound area and healing. Also, this therapeutic approach decreased the local expression of genes for proinflammatory mediators and increased the expression of the μ-opioid receptor and the MT1 and MT2 receptors in the wound area and spinal cord, with a consequent increase in reaction times in behavioural testing. *Conclusions:* In conclusion, the presented results of our study allow evidence for the advantages of the simultaneous employment of HBO and antioxidant supplementation in the treatment of thermal skin injuries, with special reference to the attenuation of painful sensations accompanied by this type of trauma.

## 1. Introduction

Thermal skin injuries are an essential and very common cause of skin damage, potentially leading to severe morbidity and significant mortality [1]. In addition to their enormous medical importance, burns also represent a major economic and social problem, considering extended hospitalization, complex rehabilitation, and expensive wound and scar treatment [2]. According to the World Health Organization (WHO), thermal burn injuries occur with a frequency of approximately 6.6 million people and are responsible for more than 300,000 deaths each year worldwide [3]. Although representing a global health problem, the vast majority of burns occur in low- and middle-income countries, mostly due to a lack of education and access to medical care [3]. The severity of the burn largely depends on the depth of the affected tissue, by which we distinguish between superficial; first-degree burns that involve only the epidermis; partial-thickness or second-degree burns that can be further divided into superficial burns, which involve the papillary dermis, and deep partial-thickness burns that extend into the reticular dermis; and full-thickness or third-degree burns extending through the entire thickness of the skin. Fourth-degree burns, aside from the entire skin, involve the underlying structures [4]. The local characteristic of a burn was described by Jackson in 1947 as a zone of coagulation—a point of maximum irreversible damage surrounded by an area of stasis—that has potentially salvageable tissue and is bordered by erythema (hyperemia) [5]. The healing of a burn wound is determined by the successive occurrence of four phases: haemostasis, inflammation, proliferation, and remodelling [6]. A few days after the injury, granulation tissue begins to form through noticeable fibroblastic proliferation. Fibroblasts are the main cell type of the dermis and are responsible for the production of essential structural proteins of the extracellular matrix, such as glycosaminoglycan and collagen. Mediators from the extracellular matrix mobilize the migration and proliferation of keratinocytes, which are major cell components of the epidermis, which initiates re-epithelisation of the wound. The epithelisation process is enabled by mitosis of the epithelial cells on the periphery of the wound and from basal cells in underlying skin appendages in partial skin-thickness wounds [7].

Pain is considered to be one of the most striking symptoms of a burn injury. Painful sensations are caused by the direct effect of thermal injury of the tissue and the stimulation of nociceptors, as well as the production and secretion of numerous mediators of inflammation [8]. The ratio of inhibitory and excitatory influences at a certain level of the CNS, including the spinal cord, brainstem, medulla oblongata, and cortical regions, largely determines the pain transition [9]. Melatonin receptor 1 (MT1) and melatonin receptor 2 (MT2), along with the main opioid receptors, play important roles in antinociceptive actions and tactile allodynia, as shown in previous research [10]. The main opioid receptors, μ (MOR) and κ (KOR), participate in anti-inflammatory, antinociceptive, and neuroprotective influences and are expressed in a wide variety of tissues, including the central nervous system, vasculature, skin keratinocytes, and immune cells [10]. Opioid receptors expressed in the skin play an important role in wound healing and are integral to normal skin homeostasis [11].

Severe burn injuries, followed by local tissue destruction and also by systemic reactions as a consequence of the extreme, dysregulated immune and inflammatory response, which may lead to shock and even multiple organ failure, present a major challenge in the therapeutic approach [12]. A local tissue hypoxia and the activation of cascade systems as a reaction to thermal tissue trauma, with a large production of highly reactive free oxygen radicals, result in a disturbance of the oxidative status [13]. Given that the brain tissue is particularly sensitive to oxidative status disbalance, neurological disorders such as cognitive dysfunctions may also occur [14]. Maintaining the balance of antioxidants and ROS can be defined as a new target in burn wound therapies [15].

Hyperbaric oxygen therapy (HBO) is a noninvasive method that includes pure oxygen (100%) treatment under pressure that is higher than normal atmospheric pressure. The importance of oxygen in wound healing has been demonstrated in various studies [16]. Mechanisms of action for hyperbaric oxygen therapy are based on the elevation of both the partial pressure of inspired O_2_ and of the hydrostatic pressure, successfully affecting physiological disorders in post-burn conditions [17]. Most recently, besides previously demonstrated effects of HBO such as improved healing, less oedema, and less infection, investigators were able to demonstrate positive effects of HBO in the reduction of post-burn pain, as well [18]. Considering all this, it is clear that HBO therapy may be beneficial to patients with second-and third-degree burn injuries.

Antioxidants are biologically active molecules that prevent the creation of free radicals in the body and at the same time neutralize the existing ones, thus, participating in maintaining the oxidative status at the desired level [19]. A traumatic state caused by thermal injury changes the balance between free radicals and the natural scavengers of the body, primarily by producing a burst of highly reactive oxygen radicals [20]. This highlights the importance of the therapeutic targeting of oxidative stress in the treatment of burns [21]. Numerous studies implicate the stimulative effect of antioxidants on wound healing-related processes [22]. Furthermore, the reduction in oxidative stress levels is set to diminish inflammation and hypermetabolism, improving clinical outcomes [23].

*Filipendula ulmaria* (L.) Maxim (FU), also known as meadowsweet, is a herbaceous perennial from the Rosaceae family, traditionally used in many European and Asian countries for its proven antioxidative, anti-inflammatory, and analgesic properties [24]. It has been shown that the rich content of phenols, primarily flavonoids and tannins, largely determines the above-mentioned pharmacological properties [25]. Considering all this, using FU extracts in post-thermal injury treatment may result in multiple benefits.

## 2. Materials and Methods

### 2.1. Housing

Two-month-old male Wistar albino rats weighing 200–250 g were obtained from the Military Medical Academy, Belgrade, Serbia. The animals were housed in transparent cages with ad libitum access to water and food and with a 12/12 h light/dark cycle. Following a 1-week habituation period, 42 rats were randomly divided into seven equal groups (6 animals per group): CONTROL, BURNS, BURNS + FU, BURNS + HBO, BURNS + FU + HBO, HBO, and FU groups.

### 2.2. Treatment

#### 2.2.1. Thermal Skin Injury Experimental Model

In total 24 animals (groups: BURNS, BURNS + HBO, BURNS + FU, BURNS + HBO + FU, and control groups) were weighed and anaesthetized with ketamine (10 mg/kg, i.p.) and xylazine (5 mg/kg, i.p.), before trichotomy of a back area measuring approximately 3 cm^2^ (1% polyvinylpyrrolidone iodine was locally applied as antisepsis). Thermal injuries were made with a solid aluminium bar (10 mm in diameter) that was electrically driven to maintain the constant temperature of 75 °C [26]. The bar was maintained in contact with the animal skin on the dorsal proximal region for 15 s.

#### 2.2.2. Hyperbaric Oxygen Treatment (HBO) and Antioxidant Supplementation with *Filipendula ulmaria* (FU) Extract

HBO treatment was performed, as previously described [27], in a specially constructed hyperbaric chamber for rats (HYB-C 300) for 7 consecutive days (100% O_2_ at 2.5 ATA for 60 min), between 1.00 and 8.00 PM (three animals in the chamber per session), in the groups: BURNS + HBO, BURNS + FU + HBO, and HBO (starting the day after the induction of thermal injury).

Starting the day after the induction of thermal injury, antioxidant supplementation was performed, as previously described [28], with *Filipendula ulmaria* extract [24] dissolved in tap water to reach a final concentration of 100 mg/kg b.w. for seven consecutive days in the groups: BURNS + FU, BURNS + FU + HBO, and FU.

### 2.3. Behavioural Testing

Behavioural testing was performed 24 h after completing the protocols. At approximately 8 a.m., rats were placed in the testing room and allowed to accommodate themselves for 1 h before behavioural testing. A nociception assessment was performed with a hot-plate test and a tail-flick test under appropriate conditions [29]. To remove potential interfering odours, the apparatuses for both tests were cleaned with water and ethanol (70%) for each animal.

#### 2.3.1. Hot-Plate Test

The hot-plate test was conducted according to an algorithm previously defined in our lab [30]. The appliance consisted of a square metal plate measuring 43 × 43 cm and glass walls of 30 cm. Each animal was placed in the central part of the plate and the temperature was maintained at 51.5 ± 0.5 °C. The duration of the test was individual and defined by the appearance of a specific reaction to a thermal stimulus in the form of licking the hind paw, shaking the hind paw, or bouncing off the ground with all 4 limbs at the same time. To prevent burns, the test time was limited to 180 s. The parameter monitored in this test is the reaction time expressed in seconds.

#### 2.3.2. Tail-Flick Test

The tail-flick test is a nociception test in which a high-intensity heat stimulus is directed at the rat’s tail according to the procedure described by Bannon et al. [31]. The animals were placed on a raised grid and covered with an tube that was appropriately sized to disable movement. After achieving a temperature of 75 °C, a heat stimulus was placed in the middle of the tail and the reaction of the experimental animal was monitored. A strong enough stimulus was necessary to provoke the expected reaction of the animal—the tail flick. By measuring the time from the initiation of the painful stimulus to the manifested form of the expected reaction, the results of this test were quantified and expressed in seconds.

### 2.4. Tissue Sample Collection

After completing behavioural testing, the rats were anaesthetized with a combination of ketamine (10 mg/kg, i.p.) and xylazine (5 mg/kg, i.p.), and sacrificed by decapitation. Spinal cords and brains were carefully removed and hippocampi were dissected. Afterwards, tissue samples were snap-frozen at −80 °C for further analysis.

### 2.5. Histological Analysis

The wounds of all animals were excised, leaving a 5 mm margin of normal skin around the edges of the wound, and fixed in 10% formal saline for histological examination for a minimum of 7 days. After the tissues were processed, mid-wound vertical sections of each specimen were cut and further processed. Specimens were dehydrated in a graded ethanol series and xylene and were embedded in paraffin. The paraffin blocks were cut into 5–7 µm thicknesses using a microtome and then attached to adhesive slides. The tissue specimens were stained with haematoxylin-eosin and Picrosirius red staining solutions and photographed using an optical microscope (Olympus BX, Tokyo, Japan) equipped with a digital camera. For quantitative analysis of collagen, bright-field images of Picrosirius red-stained sections were captured at 20× magnification, and the positive areas were measured using ImageJ software (National Institute of Health, Bethesda, MD, USA). The analysis was performed in the dermis on 5 non-overlapping fields per section, excluding blood vessels and hair follicles. Scoring and histological analysis were performed in a blinded fashion by two independent observers. The results are presented as a mean count of area percentage or as histological scores, as previously described [32]. This score system encompasses the most relevant histological parameters. Epidermis score refers to crust characteristics, presence of epithelialisation, and presence and structural characteristics of rete ridge. The histological score grade for the dermis includes the analysis of adipose cells, inflammatory cells and fibroblasts, collagen deposition, and the formation of hair follicles. The wound contraction rate was expressed as the percentage change in the original wound area using the following formula: wound contraction rate = (original wound area − wound area on day 9)/original wound area × 100% [32].

### 2.6. RNA Isolation and Real-Time PCR Analysis

Tissue specimens were excised and snap-frozen in liquid nitrogen before homogenization. Total RNA from the specimens was extracted using TRIzol reagent (Invitrogen, Waltham, MA, USA) according to the manufacturer’s instructions. For reverse transcription, iScript Re-verse Transcription Mastermix (Bio-Rad, Hercules, CA, USA) was used. Real-time PCR was carried out using SsoAdvanced Universal SYBR Green Supermix (Bio-Rad, USA) and mRNA-specific primers (Supplementary Table S1) for IL-1β, IL-6, TNF-α, TGF-β1, TGF-β3, BAX, Bcl-2, Caspase-3, Keratinocyte growth factor (KGF), Epidermal growth factor (EGF), Vascular endothelial growth factor (VEGF), Fibroblast growth factor 1 (FGF1), Fibroblast growth factor 2 (FGF2), μ opioid receptor (MOR), δ opioid receptor (DOR), κ opioid receptor (KOR), Melatonin receptors (MT1, MT2), Neuropeptide Y (NPY) and β-actin as a housekeeping gene (Invitrogen, Waltham, MA, USA). Quantitative RT-PCR reactions were performed in the Applied Biosystems 7500 (Applied Biosystems, Waltham, MA, USA) and after data analysis, relative gene expression was calculated according to Livak and Schmittgen [33].

All research procedures were carried out according to the European Directive for the Welfare of Laboratory Animals No. 86/609/EEC, the Principles of Good Laboratory Practice, and the ARRIVE guidelines. All experiments were approved by the Ethical Committee of the Faculty of Medical Sciences, University of Kragujevac, Serbia.

### 2.7. Statistical Analysis

Statistical analysis was performed with the SPSS version 20.0 statistical package (IBM SPSS Statistics 20). The results are expressed as the means ± standard errors of the mean (S.E.M.). The parameters were initially submitted to Levene’s test for homogeneity of variance and to the Shapiro–Wilk test of normality. One-way ANOVA, followed by the Bonferroni test, was used for comparisons between the groups. The significance was determined at *p* < 0.05 for all tests. Simple linear regression and Pearson’s coefficient of correlation were used to analyse the relationships between the obtained parameters.

## 3. Results

### 3.1. Morphological and Histological Parameters of the Wound Area

**Figure 1:**
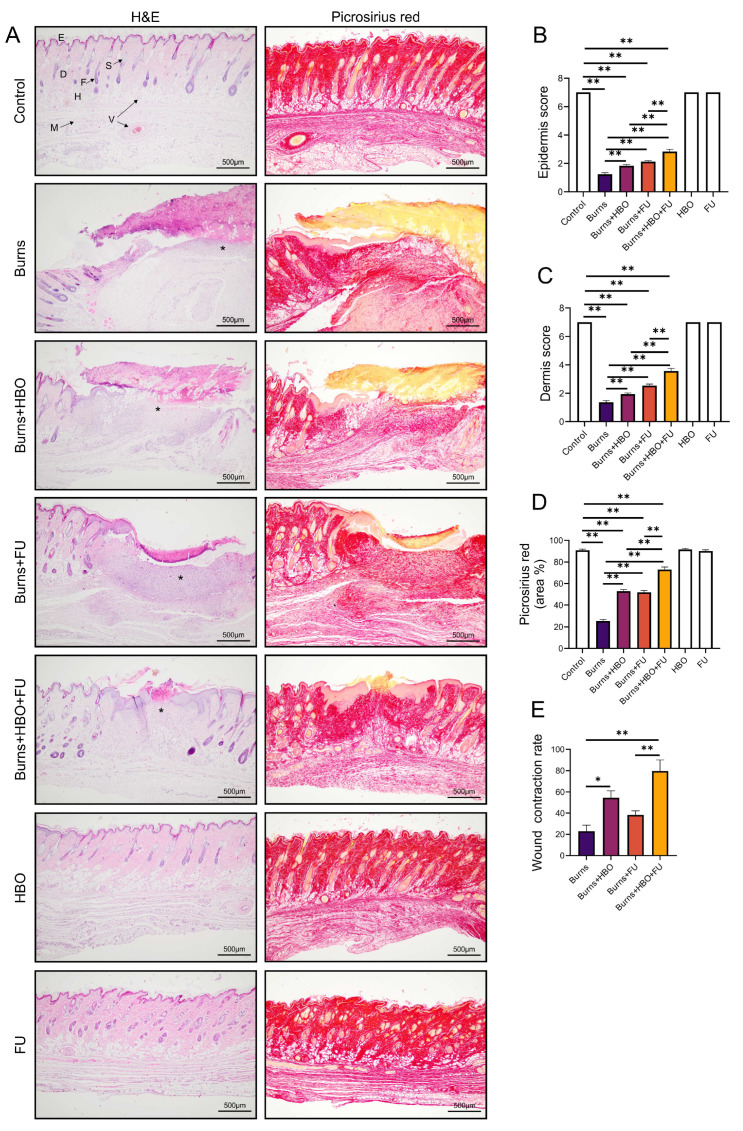
Morphological and histopathological analysis: (**A**) representative images of H&E and Picrosirius red staining on paraffin-embedded sections (original magnification 10×), (**B**) epidermis score, (**C**) dermis score, (**D**) collagen deposition, (**E**) wound contraction rate. E: epidermis, D: dermis, H: hypodermis, M: muscle fibres, F: hair follicles, S: sebaceous gland, V: blood vessel, * the initial burn regions. The values are mean ± standard error of the mean (SEM), * denotes a significant difference of *p* < 0.05; ** denotes a significant difference of *p* < 0.01.

Eight days after conducting the thermal skin injuries, we analysed the morphological and histological parameters of the wound area. The histological score for the epidermis, which shows sloughing of the crusts and subsequent re-epithelialization, was significantly lower in all experimental groups compared to the control (Figure 1A,B, F = 103,558). The obtained values in all treated groups were significantly higher compared to the burns group, while the treatment with FU extract in combination with HBO demonstrated a significantly higher re-epithelialization rate compared to FU or HBO alone (Figure 1A,B). The healing of the dermis was also analysed, and the obtained results demonstrated significantly higher scores in all treated groups compared to the burns group (Figure 1A,C, F = 699,146). The most pronounced effect was achieved with the combined FU and HBO treatment protocol, compared to FU or HBO alone (Figure 1A,C). One of the parameters of re-establishing dermal architecture, collagen formation, and deposition, was examined using Picrosirius red staining. Similarly to the dermis score, collagen content was significantly higher in all treated groups compared to the burns group (Figure 1A,D, F = 270,531). Further, the collagen content was significantly higher in the group treated with the FU and HBO combination compared to the treatment protocols alone (Figure 1A,D). In analysed histological parameters, we did not observe significant differences between groups treated with FU extract or HBO protocol alone. Macroscopic changes in wound area were presented as wound contraction rate (Figure 1E, F = 11,943). This parameter demonstrated that HBO and combined treatment with FU and HBO had beneficial effects, while there was no significant difference in the FU group compared to the burns group (Figure 1E). The wound contraction rate was significantly higher in the combined FU and HBO treatment group compared to the FU group, while there were no differences compared to the HBO group (Figure 1E).

### 3.2. Relative Expression of Genes for Proinflammatory Cytokines in the Wound Area

**Figure 2:**
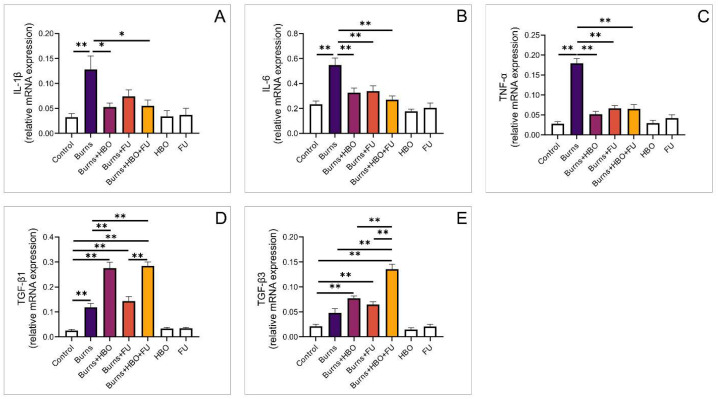
Relative expression of genes for cytokines in the wound area. (**A**) Relative expression of IL-1β; (**B**) Relative expression of IL-6; (**C**) Relative expression of TNF-α; (**D**) Relative expression of TGF-β1; (**E**) Relative expression of TGF-β3. The values are mean ± standard error of the mean (SEM), * denotes a significant difference of *p* < 0.05, ** denotes a significant difference of *p* < 0.01.

Next, we analysed the relative expression of genes for proinflammatory cytokines in the wound area. The expression of the IL-1β gene was significantly higher in the burns group compared to the control and HBO groups. Also, the expression was significantly higher in the burns group compared to the combined FU and HBO group, while there were no differences compared to the FU group (Figure 2A, F = 5477). Similarly, the relative expression of proinflammatory IL-6 and TNF-α genes was significantly higher in the burns group compared to the control group, as well as compared to all treated groups, while at the same time, there was no difference between treatment groups (Figure 2B,C, F = 10,887, F = 38,614, respectively). We further analysed the expression of genes for TGF-β1 and TGF-β3, which are cytokines involved in wound healing and tissue regeneration. Our results demonstrated that TGF-β1 and TGF-β3 gene expression was significantly higher in all treated groups compared to the control (Figure 2D,E, F = 60,922, F = 46,867, respectively). Compared to the burns group, the expression of the TGF-β1 gene was significantly higher in both groups that were treated with HBO, while there were no differences compared to FU alone (Figure 2D). Relative expression of the TGF-β3 gene was significantly higher in the group treated with HBO and FU combined, compared to burns and other treatment groups (Figure 2E).

### 3.3. Relative Expression of Genes Related to Apoptosis in the Wound Area

**Figure 3:**
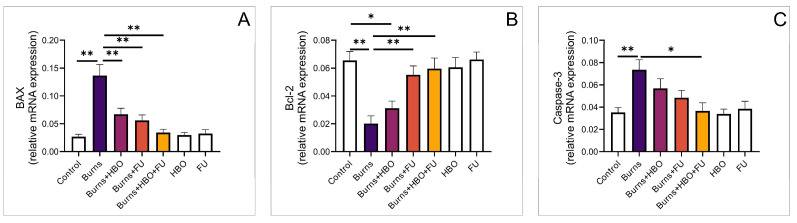
Relative expression of genes related to apoptosis in the wound area. (**A**) Relative expression of BAX; (**B**) Relative expression of Bcl-2; (**C**) Relative expression of Caspase-3. The values are mean ± standard error of the mean (SEM), * denotes a significant difference of *p* < 0.05, ** denotes a significant difference of *p* < 0.01.

Analysis of genes related to apoptosis demonstrated that expression of pro-apoptotic BAX was significantly higher in the burns group compared to the control, as well as compared to all treatment groups, while there was no difference between individual groups (Figure 3A, F = 14,432). The relative expression of the anti-apoptotic Bcl-2 gene was significantly lower in the burns group compared to the control groups treated with FU. Also, expression was significantly lower in the group treated with HBO alone compared to the control (Figure 3B, F = 8248). Relative expression of the common initiator of cell death by apoptosis, the Caspase-3 gene, was significantly higher in the burns group compared to the control and the group with combined HBO and FU treatment (Figure 3C, F = 4469).

### 3.4. Relative Expression of Genes for Growth Factors in the Wound Area

**Figure 4:**
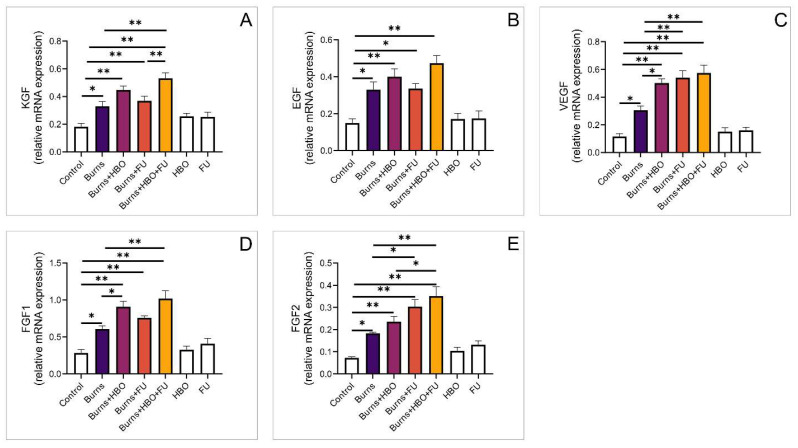
Relative expression of genes for growth factors in the wound area. (**A**) Relative expression of KGF; (**B**) Relative expression of EGF; (**C**) Relative expression of VEGF; (**D**) Relative expression of FGF1; (**E**) Relative expression of FGF2. The values are mean ± standard error of the mean (SEM), * denotes a significant difference of *p* < 0.05, ** denotes a significant difference of *p* < 0.01.

We investigated the gene expression of growth factors relevant for thermal wound healing. The expression of the KGF gene was significantly higher in all experimental groups compared to the control. In the group that received combined HBO and FU treatment, expression was significantly higher compared to the FU group and burns group, but there were no differences compared to the HBO group (Figure 4A, F = 14,882). The expression of the EGF gene was significantly higher in all experimental groups compared to the healthy control, but there were no differences between the groups (Figure 4B, F = 12,217). VEGF gene expression was also significantly higher in all groups compared to the healthy control, but it was also significantly higher in all treated groups compared to the burns group (Figure 4C, F = 30,287). FGF1 and FGF2 gene expressions were significantly higher in all groups compared to healthy controls. In animals treated with HBO, alone or in combination, the expression of the FGF1 gene was significantly higher compared to the burns group. Also, the expression of the FGF2 gene was higher, compared to burns, in the FU and combined FU and HBO groups (Figure 4C,D, F = 20,193, F = 19,321, respectively).

### 3.5. Relative Expression of Opioid and Melatonin Receptor Genes in the Wound Area

**Figure 5:**
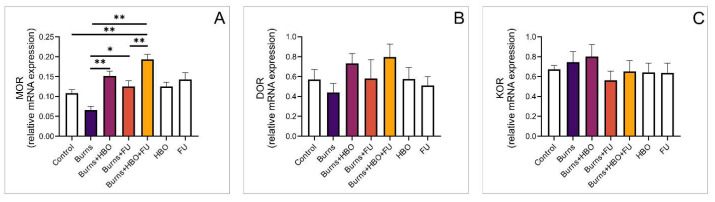
Relative expression of genes for opioid receptors in the wound area. (**A**) Relative expression of MOR; (**B**) Relative expression of DOR; (**C**) Relative expression of KOR. The values are mean ± standard error of the mean (SEM), * denotes a significant difference of *p* < 0.05, ** denotes a significant difference of *p* < 0.01.

**Figure 6:**
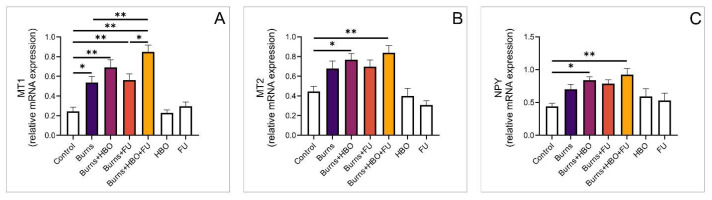
Relative expression of genes for melatonin receptors in the wound area. (**A**) Relative expression of MT1; (**B**) Relative expression of MT2; (**C**) Relative expression of NPY. The values are mean ± standard error of the mean (SEM), * denotes a significant difference of *p* < 0.05, ** denotes a significant difference of *p* < 0.01.

Further, we analysed the expression of opioid receptor genes in the skin tissue. Compared to the control group, expression of the MOR gene was significantly higher in animals that received combined treatment with FU extract and HBO. In this group, we also observed significantly higher gene expression compared to the burns and FU groups (Figure 5A, F = 10,078). Regarding the expression of DOR and KOR genes, we did not observe statistical significance among the examined groups (Figure 5B,C, F = 1054, F = 0.647, respectively). However, the expression of the MT1 gene was significantly higher in all experimental groups compared to healthy animals. Also, the expression of the MT1 gene was significantly higher in the group that received combined treatment with FU extract and HBO compared to the burns and FU groups (Figure 6A, F = 17,039). Further, the expression of MT2 and NPY genes was significantly higher in the groups with HBO alone and HBO combined with FU extract compared to the control (Figure 6B,C, F = 9725, F = 4248, respectively).

### 3.6. Relative Expression of Opioid and Melatonin Receptor Genes in the Spinal Cord

**Figure 7:**
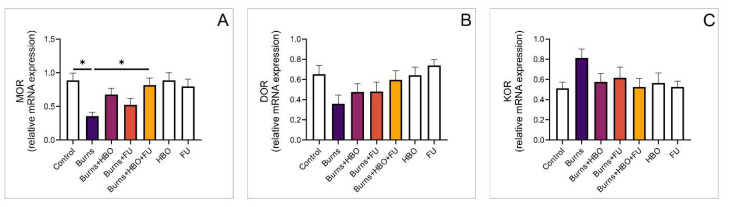
Relative expression of genes for opioid receptors in the spinal cord. (**A**) Relative expression of MOR; (**B**) Relative expression of DOR; (**C**) Relative expression of KOR. The values are mean ± standard error of the mean (SEM), * denotes a significant difference of *p* < 0.05.

**Figure 8:**
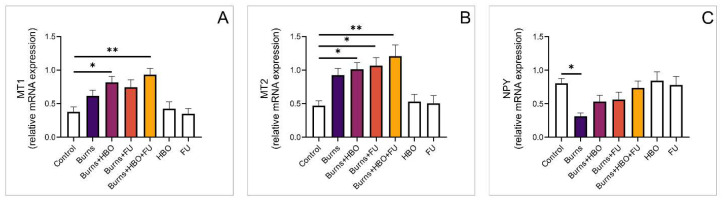
Relative expression of genes for melatonin receptors in the spinal cord. (**A**) Relative expression of MT1; (**B**) Relative expression of MT2; (**C**) Relative expression of NPY. The values are mean ± standard error of the mean (SEM), * denotes a significant difference of *p* < 0.05, ** denotes a significant difference of *p* < 0.01.

To investigate the nociceptive effects of the treatments used after a thermal skin injury, we also analysed the expression of opioid receptor genes in the spinal cord. We did not observe a significant difference between the treated groups, although the expression of MOR was significantly lower in the burns group compared to the control and combined-treatment groups (Figure 7A, F = 4198). As in the skin tissue, we did not observe a significant difference between the expression of DOR and KOR genes either (Figure 7B,C, F = 2477, F = 1525, respectively). The expression of the MT1 gene was significantly higher in animals that received HBO treatment (alone and combined with FU extract) compared to healthy controls (Figure 8A, F = 6558). Similarly, the expression of MT2 gene was also significantly higher in all treated groups compared to the control (Figure 8B, F = 7086). Relative gene expression of NPY was significantly lower in the burns group compared to the control but there was no difference compared to the other experimental groups (Figure 8C, F = 3578).

### 3.7. Behavioural Testing

**Figure 9:**
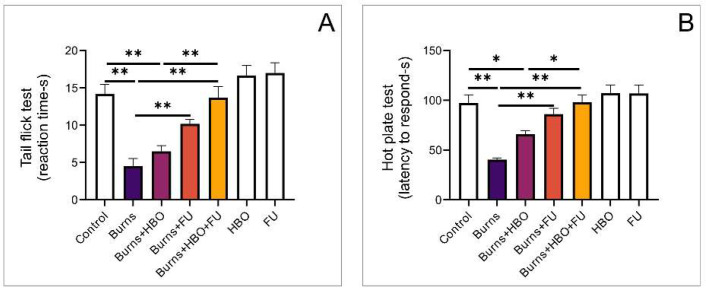
Behavioural testing: (**A**) tail-flick test, (**B**) hot-plate test. The values are mean ± standard error of the mean (SEM), * denotes a significant difference of *p* < 0.05, ** denotes a significant difference of *p* < 0.01.

The nociception assessment was performed using the tail-flick test and the hot-plate test under appropriate conditions. The results of the tail-flick test showed significant alterations in the time to respond following the thermal injury. The treatment protocol using a combination of FU extract and HBO resulted in a significant prolongation of reaction time compared to the burns and HBO-alone groups (Figure 9A, F = 17,646). Similarly, the applied thermal injury protocol significantly affected the reaction times in the hot-plate test (Figure 9B, F = 13,765). Latency to respond to thermal stimuli was significantly increased in the group with combined FU extract and HBO compared to the burns group and HBO alone (Figure 9B).

## 4. Discussion

The severity of a burn wound largely depends on the depth of the affected tissue, and healing is determined by the successive occurrence of four phases: haemostasis, inflammation, proliferation, and remodelling [6]. One of the most striking symptoms of a burn injury is painful sensations caused by the direct effect of the thermal source on the tissue and the stimulation of nociceptors, as well as the production and secretion of mediators of inflammation [8].

In the present study, we investigated the effects of FU extracts combined with HBO treatment in post-thermal injury treatment, with special reference to nociception and possible mechanisms. There is a plethora of evidence from experimental and clinical research on the topic of HBO in burn injuries, and the authors provide well-founded evidence of its beneficial interaction with the pathomechanisms of burns and healing processes [18,34]. Our findings demonstrated that combined treatment with FU extract and HBO showed significant amelioration of macroscopic as well as histopathological characteristics of the wound. Following the combined protocol, the contraction of the skin lesion was markedly increased compared to the control, as well as to orally administered FU extract alone (as presented in Figure 1). The histopathological evaluation also revealed that while there was no difference between individual applications of FU extract and HBO, there was a significant improvement when these treatments were combined. In skin sections of animals treated with combined treatment, we observed marked epithelium restoration, the presence of dense collagen fibres, and restitution of normal tissue architecture.

We can speculate that hyperbaric oxygen therapy influences the epithelialization of burn wounds by minimizing the detrimental effect of hypoxia and encouraging a faster proliferation rate and migration of epithelial cells. However, regarding this hypothesis, there are conflicting results about the role of HBO in the epithelialization of second-degree burns [35,36]. Our findings are in line with Hatibie and colleagues, who elegantly demonstrated the beneficial effects of HBO and concluded that adequate oxygen is mandatory for wound healing [37]. Although we did not measure the parameters of oxidative stress in the area of the burn injury in this study, the data we obtained are in line with recent studies that showed a positive effect of the systemic application of antioxidant therapeutics, especially on the decrease in pro-inflammatory mediators and apoptosis [38,39,40]. Furthermore, a recent study by Sheng Wu and colleagues demonstrated the upregulation of melatonin and opioid receptors after one week of HBO treatment for burn-induced pain, suggesting that they may be partly involved in the attenuation of the nociceptive sensations [10]. In our study, we demonstrated that relative gene expression of MOR and MT1 was significantly higher in the wound area compared to the control groups, as presented in Figure 5 and Figure 6.

Antioxidant supplementation during wound healing has been the subject of various investigations. It has been reported that some standard antioxidants (vitamin C [41], vitamin E [42], and selenium [43]) expressed beneficial therapeutic effects on skin injuries.

Recently, some antioxidant-rich natural products were examined for their impact on wound healing. It has been shown that some of them induce an increase in tissue repair, which is accompanied by increased levels of wound contracture, hydroxyproline, hexosamine, fibrocytes, fibroblasts, as well as better re-epithelialization, higher collagen deposition, enhanced fibronectin content, and enhanced fibroblast cells [44,45,46].

Marked changes in the hippocampal relative gene expressions were also observed for opioid receptors, which is in line with the reported downregulation of opioid receptors in the development of neuropathic pain from the perspective of the dorsal root ganglion, spinal cord, and supraspinal regions [47]. However, the alterations in opioid receptors following the previous treatments were more complex. Namely, while the significant decline in µ and δ receptor expression seven days following thermal injury was successfully abolished in the combined group, the opposite response was observed for κ receptor expression. The relative expression of κ receptors was significantly enhanced as a post-burn reaction but significantly reduced to the control values with antioxidant supplementation alone and along with HBO. The results presented in this study are in line with previously reported general upregulation of opioid receptors following a single HBO treatment in the sciatic nerve-crushed rats [48] and an even more specific increase in µ receptors after prolonged (one and two weeks) HBO treatments in rats with burn-induced neuropathic pain [10]. However, our results do not correspond to the concomitant upregulation of µ and κ receptors in the cuneate nucleus, dorsal horn, and hind paw skin that was observed following chronic HBO application [10]. A potential explanation for the differences observed in the κ receptors’ relative expression could be attributed to tissue differences for the same species, taking into account that hippocampal tissue, as the component of central analgesic mechanisms, may react differently under described circumstances.

The opioid receptors in the dorsal horn response to nociceptive stimuli were significantly altered in the opioid receptors of knock-out animals [49]. The reason for the downregulation of MOR at the dorsal horn is unclear [50,51]. The increased plasma opioids reported during the acute period of burn injury may downregulate the receptor level. This observation was probably reported because, unlike the results in our study, the samples were taken 4 weeks after the traumatic event.

The alterations in opioid/nociceptive receptors may be included in the mechanisms underlying different behavioural alterations that occur concomitantly with injuries. Thus, the changes in opioid/nociceptive receptors have been shown to influence various behavioural aspects, including anxiety and depressive state-level alterations [49,52,53] and cognitive function impairment [54], as well as alterations in nociception [53]. The beneficial role of melatonin system alterations in both peripheral and spinal cord levels was also previously reported using thermal nociceptive hypersensitivity in neuropathic rats [55]. The hypoalgesic effect of MT1 and MT2 receptors was also previously confirmed [56] and even specified for its mechanism of action via the melatonin-MT1-PKCγ pathway. As already known, MT1 and MT2 receptors are markedly present in the spinal cord [57].

The augmentation of NPY system function in the spinal cord following an injury event was previously described and reported to be accompanied by Y1 receptor internalization, and this was also confirmed for inflammation [58,59]. Furthermore, antinociceptive action via Y2 receptors, along with Y1, was also described in animal models of acute pain [60].

## 5. Conclusions

Recent research conducted on antioxidant supplementation as well as hyperbaric oxygenation in major burn trauma has been shown to improve various aspects of recovery, such as increased wound healing rates and an overall decrease in mortality. In conclusion, the presented results of our study provide evidence for the advantages of the simultaneous employment of HBO and antioxidant supplementation in the treatment of thermal skin injuries, with special reference to the attenuation of painful sensations accompanied by this type of trauma. However, as we did not observe a significant difference between the individual therapeutic options, as opposed to significant attenuation using combined therapy, further research is needed to explain the additive and mutual mechanisms of action of these therapeutic agents.

## Data Availability

Data are available upon request.

## Supplementary Materials

The following supporting information can be downloaded at https://www.mdpi.com/article/10.3390/medicina59091676/s1, Table S1: RT-PCR primers used in this study.
