# Supplementary material for: Simultaneous Administration of Hyperbaric Oxygen Therapy and Antioxidant Supplementation with Filipendula ulmaria Extract in the Treatment of Thermal Skin Injuries Alters Nociceptive Signalling and Wound Healing
Source: Medicina (Kaunas). 2023 Sep 17;59(9):1676. doi: 10.3390/medicina59091676 (PMC10536773; doi:10.3390/medicina59091676)
Supplement: Supplementary file 1 [file medicina-59-01676-s001.zip › medicina-2592021-supplementary.pdf]

Supplementary Table S1. RT-PCR primers used in this study.

	Forward	Reverse
β-actin	AAGATCCTGACCGAGCGTGG	CAGCACTGTGTTGGCATAGAGG
Bcl-2	TGTGGATGACTGACTACCTGAACC	CAGCCAGGAGAAATCAAACAGAGG
Bax	CGGCGAATTGGAGATGAACTGG	CTAGCAAAGTAGAAGAGGGCAACC
Caspase-3	GTGGAACTGACGATGATATGGC	CGCAAAGTGACTGGATGAACC
VGF	GATGACGACGACGAAGAC	CGATGATGCTGACCACAT
FGF1	ATGGCCGAAGGGGAGATCACAACC	TTAGTCAGAAGATACCGGGAGGGG
FGF-2	ACTGCCTCGAGCGGCCTGGAGATCA	CTGGTGCTAACATCAAATACGGCA
EGF	GGGAGGCTACAACTGC	GCAGCTTCCACCAACG
KGF	CAATCTAGAATTCACAGATAGGAGGAGGC	AGAATTCCAACTGCCACAGTCATGATTTC
TGF- β 1	TGGACCGCAACAACGCCATCTA	AGGCTCCAAATGTAGGGGCAGG
TGF- β 3	TGCCCAACCCCAGCTCTAAGCG	GCCTTTGAATTTGATTTCCA
IL-1 β	CACCTCTCAAGCAGAGCACAG	GGGTTCCATGGTGAAGTCAAC
IL-6	TCCTACCCCAACTTCCAATGCTC	TTGGATGGTCTTGGTCCTTAGCC
TNF-α	AAATGGGCTCCCTCTCATCAGTTC	TCTGCTTGGTGGTTTGCTACGAC
MOR	CATATTCACCCTCTGCAC	TTACAGGCAGACCGATG
DOR	TTACAGGCAGACCGATG	ATGTTTGGAATCGTCCGGTACA
KOR	TCTAGCTATTACTTCTGCATTG	TGTGTTTCTAACTCTGTTTGT
MT1	GCCACAGTCTCAAGTATGATAGG	GGTGACAAAGTTCCTGAAGTC
MT2	CCTCTACATCAGCCTCATCTGGCT	CTGCGAACATGGTTAGGAAACTGC
NPY	TCTGCCTGTCCCACCAATG	CAACGACAACAAGGGAAATGG
